# Prenylation Defects and Oxidative Stress Trigger the Main Consequences of Neuroinflammation Linked to Mevalonate Pathway Deregulation

**DOI:** 10.3390/ijerph19159061

**Published:** 2022-07-25

**Authors:** Simona Pisanti, Erika Rimondi, Elena Pozza, Elisabetta Melloni, Enrico Zauli, Maurizio Bifulco, Rosanna Martinelli, Annalisa Marcuzzi

**Affiliations:** 1 Department of Medicine, Surgery and Dentistry ′Scuola Medica Salernitana′, University of Salerno, 84081 Baronissi, Italy; spisanti@unisa.it (S.P.); rmartinelli@unisa.it (R.M.); 2 Department of Translational Medicine, University of Ferrara, 44121 Ferrara, Italy; elena01.pozza@edu.unife.it (E.P.); enrico.zauli@edu.unife.it (E.Z.); annalisa.macuzzi@unife.it (A.M.); 3 LTTA Centre, University of Ferrara, 44121 Ferrara, Italy; 4 Department of Molecular Medicine and Medical Biotechnologies, University of Naples “Federico II”, 80131 Naples, Italy; maubiful@unina.it

**Keywords:** prenylation, cholesterol, neuroinflammation, oxidative stress

## Abstract

The cholesterol biosynthesis represents a crucial metabolic pathway for cellular homeostasis. The end products of this pathway are sterols, such as cholesterol, which are essential components of cell membranes, precursors of steroid hormones, bile acids, and other molecules such as ubiquinone. Furthermore, some intermediates of this metabolic system perform biological activity in specific cellular compartments, such as isoprenoid molecules that can modulate different signal proteins through the prenylation process. The defects of prenylation represent one of the main causes that promote the activation of inflammation. In particular, this mechanism, in association with oxidative stress, induces a dysfunction of the mitochondrial activity. The purpose of this review is to describe the pleiotropic role of prenylation in neuroinflammation and to highlight the consequence of the defects of prenylation.

## 1. Introduction

The biosynthetic pathway of mevalonic acid or mevalonate is essential in both eukaryotic and prokaryotic organisms because it leads to the formation of organic compounds of enormous physiological importance, involved in many cellular processes. It is in fact an anabolic pathway that, starting from acetyl-CoA, leads to the synthesis of a family of molecules both of steroid nature, including cholesterol, and of non-steroidal nature, the isoprenoids or terpenes. Isoprenoids constitute a heterogeneous class of lipophilic molecules, being the widest family of natural molecules. They have both functional and structural properties in diverse biological processes, which range from cell membranes’ organization, gene expression regulation, post-translational modification of proteins, control of signal transduction, involvement in photosynthesis and electron transport chain, synthesis of cholesterol and its derivatives, pheromones, reproductive hormones in mammals, vitamins, and even defense against infections in plants [1,2]. Long-chain isoprenoids include ubiquinone and heme A, important for mitochondrial electron transport; the dolichol, necessary for the glycosylation of proteins; the isopentenyl group of t-RNAs; and carotenoids, which are part of the photosynthetic system of phototrophic organisms. Short-chain isoprenoids are farnesyl pyrophosphate (FPP) and geranylgeranyl pyrophosphate (GGPP), which mediate one of the most important post-translational modifications of proteins, namely prenylation. This type of ubiquitous and irreversible modification, also known as lipidation, involves the post-translational addition of hydrophobic isoprenoids to proteins and represents a crucial step as it ensures correct localization and functionality of numerous proteins essential for cellular activity. Among the prenylated proteins, there are the small proteins belonging to the family of GTP-ases, such as Ras, Rac, and Rho, as well as the nuclear laminae [3]. This modification is essential for numerous biological functions, such as cell targeting, the processes of cellular life and death (growth, differentiation, movement, autophagy), the localization of proteins in the anchoring phase to the membrane, and the regulation of their activity (protein–protein/protein–membrane interactions). As cells need a constant supply of isoprenoid compounds, they must finely tune the mevalonate pathway while avoiding excessive build-up of potentially toxic molecules, such as cholesterol itself [4]. Through numerous experimental and clinical studies, it seems that isoprenoids, essential for cell growth and differentiation, may be potential therapeutic targets in many research fields, including tumors, autoimmune diseases, atherosclerosis, and Alzheimer′s disease [5]. Moreover, an altered flux through the mevalonate pathway is involved in the pathophysiology of the Hyperimmunoglobulin D syndrome (HIDS) and Mevalonic Aciduria (MA), autoinflammatory disorders together known as mevalonate kinase deficiency (MKD) disorders, which are precisely due to a hereditary deficiency of the Mevalonate Kinase (MVK), one of the first enzymes of the mevalonate pathway [6].

## 2. The Mevalonate Pathway

**Figure 1:**
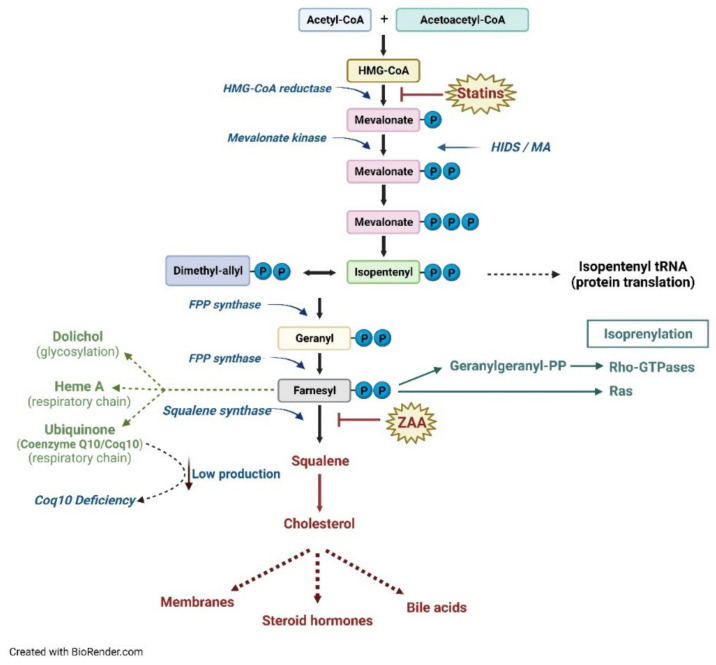
Schematic representation of the metabolic pathway of cholesterol.

The biosynthesis of the different products of the mevalonate pathway begins in the cytosol with the condensation by the thiolase of two molecules of acetyl-CoA into acetocetyl-CoA, which reacts with another acetyl-CoA molecule to form, by HMG-CoA synthase (HMGS), 3-hydroxy-3-methylglutaryl-CoA, or HMG-CoA. HMG-CoA is then reduced to mevalonic acid thanks to the action of HMG-CoA reductase (HMGR), an oxidoreductase localized in the smooth endoplasmic reticulum (ER) that uses NADPH as a cofactor (Figure 1).

Subsequently, the synthesis of mevalonate-5-phosphate by MVK occurs in the cytosol followed by the decarboxylation and transformation into a compound with five carbon atoms (C5), Δ3-isopentenyl-5-pyrophosphate (IPP), which is the basic isoprene unit for the synthesis of all other isoprenoids, such as geranyl pyrophosphate (GPP, C10), FPP (C15), and GGPP (C20), through a series of head-to-tail condensations of isoprene units catalyzed by the prenyltransferases [4]. The FPP represents the link between the synthetic pathways of non-sterols/isoprenoids and sterols. In the isoprenoid pathway, the addition of another unit of IPP to the FPP leads to the formation of GGPP. The elongation process, by incorporating further portions of IPP, generates longer isoprenoids, which have a key biological relevance, such as dolichol (essential for proteins N-glycosylation), ubiquinone (Coenzyme Q10), and heme A [7,8,9].

In particular, Coenzyme Q10 is placed in the membranes of the endoplasmic reticulum, peroxisomes and lysosomes, in the vesicles and within the membrane of the mitochondria, where it plays an important role in the electron transport chain.

The enzyme farnesyl pyrophosphate synthase (FPPS) instead catalyzes the addition of dimethylallyl pyrophosphate (DMAPP) and geranyl diphosphate to IPP to form the isomer E of FPP [10]. In the sterol branch of the metabolic pathway, in the ER, the condensation of two FPP moieties, catalyzed by the enzyme squalene synthase (SQS), produces a molecule of squalene (a molecule with 30 carbon atoms). The last stages of cholesterol biosynthesis involve the cyclization of squalene to lanosterol, which already contains the four characteristic rings of cholesterol. From lanosterol, through a series of other reactions (demethylations and isomerizations), first desmosterol and then cholesterol (at 27 carbon atoms) are formed [11] (Figure 1).

## 3. Critical Points to Regulate the Metabolic Pathway of Cholesterol

In mammalian cells, most of the mevalonate is converted into cholesterol, while the remaining mevalonate is transformed into isoprenoids; therefore, the regulation of the whole pathway is fundamental. Under physiological conditions, the levels of cholesterol and its main metabolites depend on the amount of cholesterol introduced with the diet in a homeostatic balance between processes of synthesis, absorption, transport, catabolism, and excretion. Alterations in cholesterol homeostasis, due to genetic and/or environmental factors, are thus involved in various diseases such as obesity [12], atherogenesis and cardiovascular disorders [13,14], gallstones, and some inherited neuro-metabolic diseases. In humans, the brain is the organ that presents the highest percentage of cholesterol in the whole organism, and it is located in the myelin sheath. The presence of the blood brain barrier prevents the exchange of lipoproteins and free cholesterol between plasma and cerebrospinal fluid, so that brain tissue regulates cholesterol homeostasis autonomously [15]. Consequently, cerebral cholesterol constitutes a cholesterol pool independently regulated with respect to those present in all other parts of the body [16].

The regulation of cholesterol levels, and consequently of all of the other products that derive from this biosynthetic pathway, results from the control of the HMGR enzyme, the most finely regulated enzyme of the pathway, and the rate-limiting one This mechanism is irreversible, and it represents the main system for regulating the process [17]. When HMGR sterol-sensing domain (SSD) perceives a high cholesterol content inside the cell, its conformation changes, causing enzyme proteolysis. It has been reported that several polymorphisms in the HMGR gene determine a failure of this mechanism. Such polymorphisms have been associated with statins’ efficacy, obesity, lipid metabolism, Parkinson disease, cardiovascular adverse events, and other pathologies [18,19]. Several ER proteins are able to sense cholesterol levels including HMGR, sterol regulatory-element-binding protein (SREBP), and squalene epoxidase (SQLE). A recent paper pointed to the role of mitochondrial dysfunction on the mevalonate pathway, through the reduction of pathway intermediates and downregulation of the expression of the gene pathway in an SREBP2 dependent mechanism [20]. Moreover, MVK plays an essential regulatory role in the pathway; indeed, in the two MKD pathologies, the loss of its activity causes both the accumulation of mevalonic acid and, consequently, a deficiency of the isoprenoid products downstream, demonstrating the peculiar role of this enzyme throughout the pathway [21].

The MVK enzyme is regulated at the transcriptional level in the same way as HMGR; in fact, the MVK promoter contains a sterol-regulated element (SRE) capable of inducing gene transcription following a deficit of the downstream products of the pathway (positive feedback) through SREBP2 [22]. In addition, the MVK enzyme is also subject to post-translational regulation with negative feedback by the isoprenoids GPP, FPP, and GGPP. This inhibition is of the competitive type and occurs at the binding site of the enzyme for ATP [23]. It has been recently observed that NF-E2-related factor 3 (NRF3), a transcription factor that binds ER and is involved in lipid metabolism, upregulates the expression of GGPP synthase in an SREBP2-dependent manner [24].

The study of pathologies that involve these regulatory enzymes and similarly have a serious impact on the central nervous system, such as Mevalonic Aciduria (a severe form of MKD) (OMIM#610377) [25], Smith–Lemli–Opitz syndrome (due to deficiency of 7-dehydrocholesterol-Δ7 reductase) (OMIM#270400) [26,27], Niemann–Pick syndrome (characterized by altered cholesterol trafficking and accumulation) (OMIM#257220; #607625) [28], C oenzyme Q10 deficiency (OMIM#607426) [29,30,31], made it possible to better understand the mechanisms and the regulatory systems at the basis of this metabolic pathway [32,33,34].

## 4. The Prenylation Process

Prenylation, catalyzed by a prenyltransferase, involves the addition of FPP or GGPP (with 20 carbon atoms), with the formation of a thioether covalent bond, with a thiol residue of cysteine at the C-terminal end of target proteins [35]. The bound lipid is necessary for the correct functioning of the protein itself, as it is responsible for both membrane attachment and peculiar protein–protein interactions.

**Figure 2:**
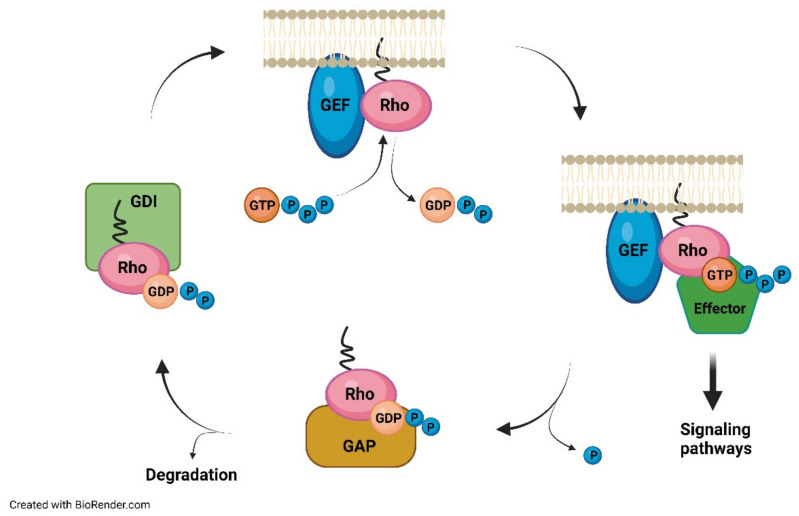
GTPase rho act as molecular switches between active and inactive GTP.

In all tissues, there are three intracellular cytosolic prenyltransferases: farnesyltransferase (FTase), geranylgeranyltransferase-I, and geranylgeranyltransferase-II (GGTase-I and II). FTase and GGTase-I are metallo-enzymes that contain a zinc atom, with 30% identity, especially in the central portion. It is unknown what the real function of zinc is in the process; perhaps it participates in catalysis, making the cysteine of the target protein more nucleophilic, or perhaps it has only a structural role [36]. FTase and GGTase-I recognize a sequence made by four amino acids, the CAAX motif in which C is a cysteine residue, A is usually an aliphatic residue, and X is specific for each enzyme. Indeed, FTase has a preference for Cys, Ala, Gln, Met, or Ser as the X residue, while GGTase-I prefers Leu, Ile, or Phe [37,38]. Genetic screening in yeast has highlighted a longer sequence target for FTase, that is, C(x)3X, expanding the list of possible human proteins that contain this motif and could thus be farnesylated [39]. GGTase-II, on the other hand, recognizes C-terminal motifs such as CC, CXC, CCX, CCXX, and CCXXX, and generally transfers GGPP to both the Cys amino acids in such sequences [40]. Prenylation increases hydrophobicity in the C-terminal domain and facilitates binding to the membrane of the ER, where the –AAX motif is cut [41]. The farnesylated and geranyl-geranylated proteins, in fact, usually are subject to a proteolytic step, catalyzed by proteases, for example, the CAAX endopeptidase 1 (RCE1), which removes the residues of –AAX downstream of the prenylated Cys [42]. The modified cysteine is then methylated by a methyl transferase, such as isoprenylcysteine carboxymethyl transferase (ICMT), to produce a protein containing a C-term farnesyl cysteine methyl ester [43]. The farnesyl group confers a weak affinity for the membrane, so other modifications are necessary for the correct localization of the proteins [44]. For example, several proteins are subject to further lipid modifications following prenylation, such as palmitoylation (palmitic acid transfer on the Cys residue with thioester bond formation) [45], useful for traffic control and anchoring to the membrane through electrostatic interactions with the anionic phospholipids positioned on the inner side of the membrane [46]. The typical targets of FTase and GGTase-I are members of the Ras superfamily, which includes a wide variety of proteins, such as Ras, Rho, and Rab, which show great functional diversification in the context of a preserved structural framework and a characteristic binding domain to GTP (Figure 2) [47].

GGTase-II, on the other hand, has a rigorous specificity for the protein substrate compared with the other two prenyltransferases; in fact, it binds with great affinity to the C-terminal residues of a complex, which also includes the Rab protein, its effective substrate [48]. All small GTPases that belong to these families must bind to membranes to activate the downstream signaling pathway, and this is possible through prenylation (or other lipid modifications) [49]. It has been recently observed in *Caenorhabditis elegans* that intracellular lipid homeostasis depends on the sequestration of the nuclear hormone receptor NHR-49 into endosomes through a specific interaction with geranylgeranylated Rab11.1. Lipid depletion, and thus a reduced flux through the mevalonate pathway, reducing Rab11.1 geranylgeranylation, induces NHR-49 translocation to the nucleus and the activation of a transcription program that leads to increased nutrient absorption [50].

## 5. Consequences Caused by Prenylation Defects

To date, it is known that prenylation defects in key enzymes of the mevalonate pathway are the basis of the pathogenesis of multiple diseases, and the two enzymes most involved in these mechanisms are FPPS and MVK [51].

FPPS is a key enzyme in the regulation of the flow of carbon atoms from the pathway of mevalonate and is responsible, among other things, for the prenylation of proteins involved in cell cycle regulation [52,53].

Among the effects of reduced FPPS activity, there is the lack of prenylation of lamin B, a protein involved in maintaining the integrity of the nuclear membrane, and the localization of the same protein in the cytoplasm. Experimental evidence suggests that G0/G1 cell cycle phase arrest is likely induced by the reduction of isoprenoid derivatives linked to the nuclear membrane protein [54].

In vitro and in vivo studies indicate that inhibition of the mevalonate pathway shows effects on the growth and progression of prostate cancer (PC) [55]. The expression of FPPS appears, in fact, increased in patients with PC and nitrogen-containing bisphosphonates, inhibitors of FPPS, represent the elective treatment for bone metastases of this carcinoma. In vitro studies on PC cells have found that bisphosphonates affect tumor invasion and angiogenesis, and that zoledronic acid, a drug belonging to this group, inhibits the survival and proliferation of cancer cells with effects that appear to be the result of a lack of prenylation of small GTPases [56].

GTPases, such as Ras, among the most frequent oncoproteins mutated in human tumors, also need to be prenylated and are localized on the inner surface of the cell membrane so that proliferation cell pathways, such as those of PI3K/Akt and Raf/Mek/ERK, can be activated [57]. In particular, in the central nervous system (CNS), the Ras farnesylation plays a crucial role in regulating synaptic plasticity and determining synapse identity, while Rho GTPase carries out neuroprotection activity [58,59].

Impaired activity of MVK leads to a reduction in the production of isoprenoid molecules and to a defective protein prenylation, with consequent cytosolic accumulation of non-prenylated proteins [60].

It has been proposed that the excessive production of IL1β observed in patients with MKD, suffering from the congenital deficiency of the enzymatic activity of MVK [61], could be caused by the loss of protein prenylation, in particular when GGPP is missing; this event favors an overactivation of the inflammasome, thus triggering the systemic inflammatory peaks characteristic of the pathology [62,63].

In in vitro experiments, MKD models were obtained by treating cells with statins (HMGR inhibitors), bisphosphonates (FPPS inhibitors), or specific GGTase inhibitors to mimic protein prenylation blocking [64]. Recently, studies conducted by Skinner et al., have clearly shown that the loss of prenylation of some GTPases, such as Rac1 or RhoA, leads to the activation of inflammasome and thus of caspase 1, with increased production of IL1β; this has been observed in both human monocytic cells treated pharmacologically with statins, and directly in cells of patients suffering from MKD, upon stimulation with LPS [65].

Other studies have shown that reduced RhoA prenylation may be the basis for the excessive production of IL1β observed in MKD [66]. In cases of isoprenoid deficiency, there is increased RhoA activity, which leads to a further increase in the gene expression of pro-IL1β, as well as the activation of Rac1, which induces pro-caspase-1 in the inflammasome [67]. Similarly, the lack of isoprenoids also impairs mitochondrial function and stability, as well as autophagic clearance of damaged mitochondria, further promoting hypersecretion of IL1β [67].

Similar mitochondrial disorders and pro-inflammatory cell death have also been observed in statin-treated neuronal cells, suggesting that these events may contribute to neurological damage observed in patients severely affected by MA [67].

The results obtained from these studies are especially important from the clinical point of view, as they could help to overcome the deficit associated with the mevalonate pathway, restoring the normal prenylation of proteins that play a fundamental role in the activation of inflammation.

## 6. Neuroinflammation, Oxidative Stress, and Fever as a Consequence of Altered Mevalonate Pathway Flux

It is well known that the process of inflammation is mediated by the cells of the immune system and by specific chemical factors such as pro-inflammatory molecules; once the damaging agent is recognized, leukocytes and proteins are called back through chemical mediators from the bloodstream to the damaged site, where, once activated, they intervene in different ways [68]. Initially, monocytes/macrophages are the first cell population that releases pro-inflammatory cytokines and chemokines and induce phagocytosis [69]. Neuroinflammation plays a fundamental role in the CNS, exerting both possible beneficial and harmful effects on nervous tissue: a mild and rapid inflammatory state has a neuroprotective action, while the presence of a chronic inflammatory process could lead to negative effects [70]. As in the case of classical inflammation, at the nervous system level, it is also possible to make a distinction between acute neuroinflammation, which is basically a defensive response of the body to a harmful insult, resulting in repair of the damaged site, and chronic neuroinflammation, characterized by persistent damaging stimuli, which can result in neurodegeneration [71].

The neuroimmune system plays a particularly important role because it is involved in normal functioning, development, and aging, and intervenes in the case of CNS lesions. The homeostasis of this anatomical area is based on the good functioning of the blood–brain barrier and the presence of a large variety of cells: neurons, astrocytes, oligodendrocytes, pericytes, and microglia cells interact with each other, and their activity is essential for a multitude of brain functions [72,73].

Microglia cells represent 5–12% of all cells present in the CNS and play the fundamental role of the first line of defense of the CNS [70,74]. The essential role attributed to microglia cells is the sentinel function, which is the ability to constantly detect changes in their environment; the cleaning function, which promotes neuronal well-being; and the aforementioned defense function, providing neuroprotection [75]. Microglia cells are involved in maintaining CNS homeostasis; controlling synaptic density, connectivity, and plasticity; eliminating myelin debris and apoptotic cells; and affecting germination, migration, anastomosis, and improvement of the increasing vascularization of the CNS [76].

In addition, these cells, as all the other macrophages present in the body, perform phagocytosis, activate cytotoxicity mechanisms, and contribute to the inflammatory response through the production of signal molecules [77]. However, prolonged activation of microglia involves the acquisition of a harmful phenotype, with the release of inflammatory mediators that promote protein aggregation and neuronal damage [78].

An imbalance of these microglial functions may trigger the onset or exacerbation of neurodegeneration, a severe and debilitating neuroinflammatory disease that may occur as a result of specific and persistent stimuli, with progressive degeneration and death of neurons. All of this is because of microglia cells, which could also damage and kill neurons, depending on the type of inflammatory response, resulting in psychomotor damage, which characterizes the phenotype of neurodegenerative diseases [79]. Noteworthy, MKD syndromes are characterized, among the other clinical features, by recurrent episodes of fever, one of the clinical signs that unites autoinflammatory diseases with other inflammatory symptoms and neurological involvement especially in the most severe forms (mental and psychomotor retardation, progressive cerebellar ataxia, visual impairment, epilepsy) [6]. Fever represents an adaptive, temporary, and reversible reaction, implemented systematically by the body in response to an inflammatory stimulus that can be caused by substances, called pyrogens, of both the exogenous kind, such as viruses, bacterial agents, and their products, or the endogenous kind, such as various cytokines and pro-inflammatory molecules [80].

From a physiopathological point of view, fever is the result of the action of prostaglandin E2 (PGE2), a metabolite of arachidonic acid, which acts on the thermoregulatory center of the hypothalamus [81]. As a result of the phlogistic insult, pyrogen cytokines, such as IL1β, IL6, and TNFα, are produced by macrophages; they interact indirectly on the thermoregulatory neurons of the hypothalamus, because they stimulate the endothelial cells of the hypothalamic vessels to produce PGE2, which in turn acts on the neurons. Finally, the concentration of cyclic AMP (cAMP) at the hypothalamic level increases and the body temperature increases above the threshold [82].

In general, oxidative stress is a condition in which there is an imbalance between the production of reactive oxygen species (ROS) and the action of antioxidant defenses [83]. Overproduction of ROS leads to progressive damage to cellular molecules, such as DNA, and a mitochondrial dysfunction that in turn generates a further increase in ROS production, compromising cell integrity and viability. Brain cells, in particular, are very sensitive to the effects of oxidative stress and, in such conditions, microglia and astrocytes are stimulated to release inflammatory mediators such as iNOS and to trigger cyclooxygenase 2 (COX-2), causing a neuroinflammatory response [84,85,86].

In particular, oxidative stress in neuroinflammation is a process characterized by the activation of the glia, which underlies a continuous cycle of inflammatory events with the release of cytokines and other neurotoxic mediators [84].

To understand the role of oxidative stress in the biogenesis of neuroinflammation, it should be considered that the inflammation at the molecular level sees the involvement of inflammasome, a multi-protein complex to which NLRP1, NLRP3, NLRP6, and NLRPC4 belong. These proteins are part of the super-family of cytoplasmic receptors called NOD-like receptors (NLR) and are activated in the presence of molecular patterns associated with pathogens (PAMPs) or stress/cell damage (DAMPs) [87,88]. One of the best known components is the NLRP3-inflammasome, which is able to recruit and activate the pro-inflammatory caspase 1, belonging to a family of molecules responsible for the apoptotic cell process. Activated caspase-1, in turn, allows the activation of three pro-inflammatory cytokines: IL-1β, IL-18, and IL-33.

NLRP3-inflammosoma also induces the activation of nuclear factor kB (NF-kB), the main orchestrator of gene transcription during the inflammatory process, the resolution phase of which occurs through a particular form of apoptosis, called pyroptosis [89]. The delicate balance between these two forms of programmed death is fundamental in sustaining inflammation, both systemic and nervous, through the pathway dependent on caspase-9, further confirming the mitochondrial involvement in these processes [90].

In this regard, several literature data have shown that an impaired mitochondrial function is associated with the release of ROS or nitric oxide (NO), which in turn determines the activation of inflammasome [91,92,93].

Finally, in the pathogenesis of neurodegenerative diseases, NLRP3-inflammasome plays a crucial role, also thanks to the fact that the literature data indicate that it is expressed in the cells of the immune system and in the CNS [94,95].

Mitochondrial damage represents a pivotal event of apoptosis in response to various conditions of intracellular stress (DNA damage, cytotoxic damage, oxidative stress, and infections). These stimuli act by inhibiting or activating members of the Bcl-2 family, such as Bak, Bax, Bad, Bcl-xl, and Bim [96]. These pro-apoptotic proteins play a fundamental role because, in the presence of overproduction of ROS, they induce the formation of channels in the mitochondria, as reported in Coenzyme Q10 deficiency [97,98]. Recent landmark works have demonstrated that both a limited as well as a permanently increased flux through the mevalonate pathway trigger alarms and lead to distinct inflammatory and immune responses [99,100]. Increased levels of brain isoprenoids FPP and GGPP have been detected in hyperglycemia, where RhoC is induced in the liver by proinflammatory cytokines and in male Alzheimer patients [101,102]. These sex-dependent alterations in the mevalonate flux have been reported in both the liver and brain, where marked differences in regions involved in memory and learning functions could be at the basis of clinically relevant differences among males and females in both neurodevelopmental and neurodegenerative diseases [103]. Interestingly, FPP has been recently reported to act as a danger signal in the brain, inducing neuronal death in a mouse stroke model through the activation of transient receptor potential melastatin 2 [104]. In diseases associated with prenylation defects such as MKD, the typical neurodegeneration has been reported to be linked to both caspase-9/3-dependent apoptosis, triggered by mitochondrial damage, and to pyroptosis mediated by caspase-1, which in turn activates cytokines and pro-inflammatory chemokines, playing a crucial role in neuroinflammatory mechanisms [90,105,106].

The overproduction of IL-1β in MKD syndromes, linked to neuroinflammation, fever, and oxidative stress, is thus considered a causal factor and is the reason anti-IL-1 therapeutic approaches (anakinra, canakinumab) have been approved for MKD treatment and have been reported to be at least partially effective in some patients [25,61,107,108].

## 7. Coenzyme Q10: The Fine Regulation of Its Antioxidant Properties

Coenzyme Q10 (Coq10), also improperly called vitamin Q, is a lipid-soluble molecule with powerful antioxidant properties, identified for the first time only in 1957, and produced naturally by the human organism in which it has an ubiquitous distribution, that is, it is present in all its cells [109]. Coenzyme Q10 is located in particular in cell membranes and mitochondria, highly differentiated structures present in the cytoplasm of plant and animal cells with aerobic metabolism. It plays a decisive role in the synthesis of ATP at the level of the electron transport chain in the mitochondria, allowing the production of ATP, and thus of energy; without the key role of Coq10, the chain would be interrupted by preventing the production of ATP.

As mitochondria are present in greater numbers in tissues characterized by a particularly active oxidative metabolism, such as the heart, brain, liver, pancreas, skeletal muscles, and brown adipose tissue, the role of Coq10 is particularly crucial in these anatomical districts. Moreover, Coq10 protects LDL (low-density lipoprotein), sometimes called “bad” cholesterol, from oxidation, and oxidized LDL is particularly harmful because it triggers inflammatory processes in the blood vessels, contributing to the creation of atherosclerotic plaques [110,111,112].

Thanks to these properties, supplements of Coenzyme Q10 are proposed in the case of deficiency or from a prevention perspective, used as nutraceutical, in heart disease, hypertension, neurodegenerative pathologies, cellular aging, and photo-aging. In addition, the integration of Coq10 has also been proposed as a support to drug therapies, and as protection from oxidative stress in the case of intense exercise, for the reduction of fatigue, and improvement of sports performance.

Coq10 supplementation is often associated with the pharmacological treatment of statins. Statins, in fact, are generally very well tolerated, but can induce some kind of muscle toxicity, characterized by various clinical manifestations, and the main reason is associated with the biochemical function of statins as a hypocholesterifying that cause a reduction in the physiological level of Coq10. Statin myopathy includes muscle weakness or pain (myalgia), hypersensitivity and muscle stiffness, cramps, and arthralgia, and it is detected by plasma creatine kinase levels [113,114]. The prevalence of this complication in patients treated with statin varies between 7% and 29%; this muscle toxicity is caused by an accumulation of statin in myocytes and can be caused by defects in the metabolism of the statin, as well as by muscle factors such as mitochondrial damage and production of ROS.

Coq10 has been shown to be very useful in counteracting these effects, as it has a myo-protective action and promotes muscle well-being. In addition, it helps to reduce the sense of fatigue and it is essential to maintain a good physical efficiency and a proper cellular metabolism [115,116].

Symptomatology resulting from a Coq10 synthesis is an induced and transient phenomenon that must be distinguished from rare genetic neurometabolic disease, referred to as Coq10 deficiency (OMIM #607426, #614652). It is an autosomal recessive transmission disease and the damage caused by this condition can affect all organs; however, depending on the form of the pathology, the organs more affected are the kidneys, cerebellum, skeletal, and heart muscles [117].

When the kidneys are involved, nephrotic syndrome is established, which can lead to renal failure [118,119]. If the cerebellum is mainly affected, the disease manifests itself with difficulty in walking and coordinating movements or convulsions [120,121], while the third form affects the muscles and manifests itself with muscle weakness [117,122]. Typically, symptoms appear during childhood, but may also occur later. To date, the best pharmacological treatment for this pathology is represented by the oral administration of Coq 10.

The study of the wide-ranging effects of this deficit has made possible the understanding of the antioxidant and protective role played by Coq10.

## 8. Conclusions

The manipulation of the metabolic pathway of cholesterol by inhibitors is aimed at regulating the synthesis of the final product. The blocking of activity of the HMG-CoA reductase and the FPP by inhibitors is focused on acting specifically at different levels of the pathway to counteract the overproduction of cholesterol. The mechanisms implemented by these compounds, which are the basis of the pharmacological active principles commonly used in the treatment of hypercholesterolemia such as statins and bisphosphonates, have highlighted transduction pathways′ signals triggered by the block of the metabolic pathway. One of the most important regulatory mechanisms is undoubtedly the reduction of regulatory proteins that bind GTP through the blocking of the production of farnesyl-pyrophosphate. The main substrates of post-translational prenylation modifications are represented by G proteins such as Rho and Rac, which function as molecular switches and, through the transduction of extracellular signals, act on cell survival, growth, and programmed cell death. These inhibitors also act at the endothelial level through the upregulation of nitric oxide synthase, and this system is certainly the basic mechanism to counteract the formation of atherosclerotic plaque. The prenylation defects are considered responsible for the inflammatory process owing to the deficiency of the intermediates of the metabolic pathway such as isoprenoids. This condition is the basis of the pathogenesis of rare pediatric diseases such as MKD, the most severe form of which experiences a significant state of neuroinflammation and clinically manifests itself with a psychomotor delay in patients. The neuroinflammatory response is characterized by a series of changes that mainly involve the role of microglial cells in the maintenance of cerebral homeostasis. An imbalance at this level involves activation, by specific brain mediators, of microglia that is in turn responsible for amplifying and maintaining the inflammatory state. Moreover, oxidative stress has been shown to be one of the main causes of mitochondrial dysfunction, resulting in alteration of ATP production, as in Coq10 deficiency.

The effects of prenylation deregulation and oxidative stress converge to determine specific morphological changes, especially on the mitochondrial compartment, and the deepening of the molecular mechanisms underlying these modifications is at the heart of considerable scientific interest as the potential spillovers of such knowledge are aimed at identifying transversally innovative therapeutic targets.

## Data Availability

Not applicable.
